# Phase I Trial of Upamostat Combined With Gemcitabine in Locally Unresectable or Metastatic Pancreatic Cancer: Safety and Preliminary Efficacy Assessment

**DOI:** 10.1002/cam4.70550

**Published:** 2024-12-30

**Authors:** Xiuping Lai, Di Cheng, Huixin Xu, Jingshu Wang, Xiaozhi Lv, Herui Yao, Liuning Li, Junyan Wu, Suiwen Ye, Zhihua Li

**Affiliations:** ^1^ Phase I Clinical Trial Center Sun Yat‐sen Memorial Hospital, Sun Yat‐sen University Guangzhou China; ^2^ Department of Medical Oncology Sun Yat‐sen Memorial Hospital, Sun Yat‐sen University Guangzhou China; ^3^ Department of Medical Oncology GuangDong Province Hospital of Chinese Medicine, the Second Clinical Medical Collage, University of Guangzhou Traditional Chinese Medicine Guangzhou China

**Keywords:** LH011, maximum tolerated dose, pancreatic cancer, upamostat, urokinase inhibitor

## Abstract

**Aim:**

This study aimed to determine the maximum tolerated dose (MTD) of the urokinase plasminogen activator (uPA) inhibitor upamostat (LH011) in combination with gemcitabine for locally advanced unresectable or metastatic pancreatic cancer.

**Method:**

Seventeen patients were enrolled and received escalating doses of oral LH011 (100, 200, 400, or 600 mg) daily alongside 1000 mg/m^2^ of gemcitabine. Safety profiles, tumor response (including response rate and progression‐free survival), pharmacokinetics, and changes in CA199 and D‐dimer levels were assessed.

**Results:**

During the study period (Day0–Day49), no patients achieved partial response. Stable disease (SD) was observed in 12 patients (70.6%), while four patients (23.5%) experienced progressive disease (PD). One patient withdrew due to a serious adverse event (SAE) on D47. Pharmacokinetic analysis revealed a dose‐related increase in LH011 and its metabolite WX‐UK1 exposure from 100 to 400 mg but not in the 600 mg group. Hematological toxicity, mainly attributable to gemcitabine, was the predominant grade 3 or 4 adverse event, with additional occurrences of loss of appetite, rash, and interstitial lung disease. Sinus bradycardia possibly linked to LH011 rather than gemcitabine was noted. The MTD was not reached.

**Conclusion:**

Combining LH011 at doses ranging from 100 to 600 mg with gemcitabine every 21 days demonstrated manageable safety and tolerability. However, tumor response did not significantly differ among the dose groups, suggesting the need for further investigation.

**Trial Registration:**

NCT05329597

## Graphical abstract

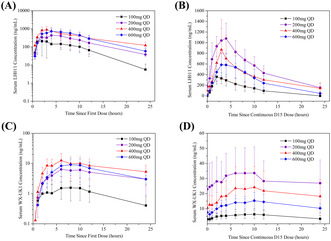

## 1 Introduction

Pancreatic ductal adenocarcinoma (PDAC) is a significant contributor to cancer‐related mortality and is projected to become the second leading cause of cancer‐related death by 2040 [1]. A key challenge contributing to the high mortality rate of PDAC is its often asymptomatic nature, leading to late‐stage detection [2]. Despite advancements in targeted therapies, chemotherapy remains the primary treatment for advanced PDAC. Since 1997, Burris and colleagues demonstrated the survival benefits of gemcitabine (GEM) monotherapy in inoperable advanced PDAC, which continues to be a cornerstone of chemotherapy regimens [3].

Although gemcitabine‐based therapies are widely used, clinical studies have shown limited median progression‐free survival (PFS) and overall survival (OS) rates for metastatic PDAC patients, highlighting the need for more effective treatment options [4, 5]. Notably, the FOLFIRINOX regimen has shown the longest median OS and PFS duration. However, its significant toxic side effects limit its widespread application [6]. The classical MPACT trial demonstrated that the combination of gemcitabine and nab‐paclitaxel (AG regimen) is more effective than gemcitabine monotherapy in patients with metastatic PDAC who have not received prior systemic therapy. While the AG regimen showed improvements in both PFS and OS, the survival outcomes remain suboptimal [7]. So there remains an urgent need to identify adjuvants that can enhance the efficacy of gemcitabine‐based treatments.

The urokinase‐type plasminogen activator (uPA) system plays a crucial role in tumor invasiveness and metastasis across various cancers, including PDAC [8, 9]. Elevated levels of pretreatment serum uPA have been associated with poorer survival outcomes in advanced PDAC patients [10]. Inhibitors of uPA have shown promise in reducing tumor invasiveness and metastases.

Upamostat (LH011) represents a potential therapeutic avenue as an oral prodrug of the serine protease inhibitor WX‐UK1. Preclinical studies have demonstrated the efficacy of WX‐UK1 in reducing tumor growth and metastasis in animal models. In a phase II trial, the combination of upamostat and gemcitabine showed promising efficacy and safety profiles in advanced PDAC patients [11]. Here, we present the results of the efficacy and safety of upamostat plus gemcitabine in advanced and metastatic PDAC patients from the phase I trial of upamostat.

## 2 Patients and Methods

### 2.1 Patient Selection

In this study, we investigated the safety and efficacy of the uPA inhibitor upamostat in combination with gemcitabine for patients with advanced or locally advanced unresectable pancreatic cancer. Eligible participants had histologically or cytologically confirmed pancreatic adenocarcinoma, were aged between 18 and 75 years, had an Eastern Cooperative Oncology Group (ECOG) performance status of 0–2, a life expectancy of at least 3 months, and demonstrated adequate bone marrow, liver, kidney, and coagulation function. Measurable disease based on RECIST v1.1 criteria was also required [12].

Patients were excluded if they had significant coagulation or hemorrhagic disorders, recent anticoagulant or thrombolytic therapy, or had received anti‐tumor treatments within 4 weeks prior to the study. Additionally, participants must have had resolution of any common toxicities from prior therapy to grade 1 or 0 (except for alopecia). Patients with brain metastases or spinal cord compression without surgical or radiation intervention were eligible, while those with gastrointestinal abnormalities, active uncontrolled infections, significant cardiac insufficiency or hepatopathy, a history of nephrotic syndrome, HIV positivity, pregnancy, breastfeeding, or inability to use appropriate contraceptive measures were excluded.

### 2.2 Study Design and Treatment

This phase I study, a nonrandomized, single‐arm, dose‐escalation trial, aimed to determine the maximum tolerated dose (MTD) of LH011 in combination with gemcitabine, assess its initial efficacy, and evaluate its impact on CA19‐9 and uPA‐related markers such as D‐dimer.

LH011 was administered orally at escalating doses of 100, 200, 400, and 600 mg once daily for 28 days, with 240 mL of water under fasting conditions. This regimen included a 7‐day period of LH011 alone followed by 21 days of LH011 plus GEM. On Days 8 and 15, gemcitabine was added at a dose of 1000 mg/m^2^. Subsequent cycles consisted of 21‐day periods, with LH011 administered daily and gemcitabine added on Days 1 and 8. Tumor evaluation occurred after 2 cycles, and patients showing nonprogressive disease continued treatment with informed consent until disease progression or intolerance to toxicity.

A standard 3 + 3 dose‐escalation method, as described by Eisenhauer et al. [13] was employed. Three patients were enrolled in each dose cohort. If no dose‐limiting toxicity (DLT) was observed in the first 28 days, the cohort advanced to the next dose level. If one patient experienced DLT, three more patients were enrolled; if two patients experienced DLT, enrollment to that dose level was halted, and the MTD was determined as the lower dose.

### 2.3 Assessments

#### 2.3.1 Tumor Assessment

Baseline tumor assessment must occur within 28 days before enrollment. The initial evaluation follows the first gemcitabine administration at 6 weeks. If there is no disease progression and the investigator approves continuation, the patient may consent to maintain the original regimen, undergoing subsequent tumor evaluations every 6 weeks. Assessment adheres to RECIST version 1.1 guidelines [11].

#### 2.3.2 Safety and Pharmacokinetic Assessments

Upamostat (LH011) is an oral prodrug of the serine protease inhibitor WX‐UK1, which upon absorption, is metabolized to its active, noncytotoxic form, WX‐UK1.

An adverse event (AE) is defined as any new undesirable medical experience or a significant alteration of a pre‐existing condition that occurs during or after the administration of the investigational agent, regardless of its causal relationship to the agent. Adverse events (AEs) will be documented starting from the time the patient signs the informed consent form.

DLTs refer to AEs that occur within the first 28 days of treatment and meet the following criteria: Grade 4 neutropenia lasting more than 7 days; febrile neutropenia (absolute neutrophil count < 1.0 × 10^9^/L with a single episode of fever ≥ 38.3°C or fever ≥ 38°C lasting more than 1 h); Grade 4 neutropenia associated with infection; Grade 4 thrombocytopenia lasting more than 48 h or accompanied by increased bleeding; and Grade 4 anemia. Additionally, Grade 3 or higher nonhematologic AEs will be included.

Blood samples were collected to assess the pharmacokinetic (PK) profiles of upamostat, WX‐UK1, and gemcitabine. Following the initial LH011 dose, blood samples were obtained at 0 (within 30 min prior to administration), 0.5, 1, 2, 3, 4, 6, 8, 10, 12, and 24 h for PK analysis. During the LH011 plus gemcitabine period, blood samples were collected on Day 15 to assess the steady‐state concentration of LH011 at the same time points.

## 3 Results

### 3.1 Patient Characteristics

A total of 17 patients were enrolled in the study between May 2020 and November 2022, all receiving at least one dose of LH011: 100 mg (*n* = 3), 200 mg (*n* = 3), 400 mg (*n* = 6), and 600 mg (*n* = 5). Sixteen patients completed 2 cycles of administration and a 49‐day follow‐up. Following this, six patients opted to continue treatment under medical consent due to stable tumor assessments (see Table 1).

**TABLE 1:** Enrollment and treatment.

Treatment Arm	Enrolled	Withdrawal	Finished D49	Continue the treatment
Arm A: 100 mg LH011 + GEM	3	1	2	1
Arm B: 200 mg LH011 + GEM	3	0	3	1
Arm C: 400 mgL H011 + GEM	6	0	6	2
Arm D: 600mgLH011 + GEM	5	0	5	2

Patient baseline characteristics are summarized in Table 2. The median age was 62 years (range, 43–70 years), with five female and 12 male patients. ECOG performance status ranged from 0 to 1, and 12 patients (70.6%) had prior chemotherapy experience, including seven patients (41.2%) who had received gemcitabine.

**TABLE 2:** Demographic and baseline clinical characteristics for pancreatic cancer.

Characteristic	No of patients (*n* = 17)	%
Age, years		
Median	62	
Range	43–70	
Sex		
Female	5	29.4%
Male	12	70.6%
No. of prior treatments		
1	7	41.2%
2	6	35.3%
3	3	17.6%
4	1	5.9%
ECOG performance status		
0	2	
1		
Previous treatment		
Prior surgery	10	58.8%
Prior chemotherapy	12	70.6%
With gemcitabine	7	41.2%
CA19‐9 baseline, mg/l		
Median	897	
Range	5.2–> 10,000	

### 3.2 Escalation, DLT, and Maximum‐Tolerated Dose

Based on early clinical studies of LH011, data indicated that once‐daily doses of 200 or 400 mg of LH011 in combination with gemcitabine were safe and well‐tolerated. Considering potential differences in body size between Chinese and Western populations, the initial dose for this regimen was set at 100 mg once daily. Notably, no DLTs were observed at doses of 100, 200, or 400 mg once daily.

However, three serious adverse events (SAEs) occurred at the 100 and 200 mg doses beyond the DLT observation period. Consequently, three additional patients were enrolled at the 400 mg dose level, which was also well‐tolerated. Subsequently, the dose was escalated to 600 mg, where DLTs remained absent. Therefore, the MTD was not established within this protocol.

### 3.3 Safety

Treatment‐related AEs were observed in all 17 patients (100%), as detailed in Table 3. The most common AEs were hematological, including leucopenia, neutropenia, thrombocytopenia, and anemia. Other notable AEs, mostly grade 1 or 2, included nausea, vomiting, diarrhea, constipation, fever, fatigue, peripheral edema, elevated glutamic‐pyruvic transaminase (ALT), elevated glutamic oxalacetic transaminase (AST), and elevated D‐dimer. These AEs were attributed to both gemcitabine and LH011. The primary grade 3 or 4 events were hematological toxicities, as expected with gemcitabine treatment. Additionally, loss of appetite (1/17, 5.9%), rash (2/17, 11.8%), and interstitial lung disease (2/17, 11.8%) were observed. These AEs align with findings from previous study [11]. Notably, sinus bradycardia was possibly linked to LH011 rather than gemcitabine, leading to one patient's withdrawal from the study.

**TABLE 3:** Treatment‐emergent adverse events.

	Arm A: (*n* = 3)		Arm B: (*n* = 3)		Arm C: (*n* = 6)		Arm D: (*n* = 5)	
Category/adverse event	All grade (%)	Grade 3/4 (%)	All grade (%)	Grade 3/4 (%)	All grade (%)	Grade 3/4 (%)	All grade (%)	Grade 3/4 (%)
AE patients	3 (100.0)	2 (66.7)	3 (100.0)	3 (100.0)	6 (100.0)	4 (66.7)	5 (100.0)	3 (60.0)
Constitutional								
Fever	1 (33.3)	0	2 (66.7)	0	3 (50.0)	0	1 (20.0)	0
Fatigue	0	0	3 (66.7)	0	2 (33.3)	0	1 (20.0)	0
Edema peripheral	0	0	1 (33.3)	0	2 (33.3)	0	2 (40.0)	0
Loss of appetite	1 (33.3)	0	1 (33.3)	1 (33.3)	2 (33.3)	0	1 (20.0)	0
Hematological/laboratory								
Leucopenia	2 (66.7)	1 (33.3)	3 (100.0)	1 (33.3)	4 (66.7)	3 (50.0)	4 (80.0)	0
Neutropenia	3 (100.0)	2 (66.7)	2 (66.7)	1 (33.3)	4 (66.7)	2 (33.3)	4 (80.0)	1 (20.0)
Thrombocytopenia	1 (33.3)	1 (33.3)	2 (66.7)	1 (33.3)	5 (83.3)	1 (16.7)	3 (60.0)	1 (20.0)
Anemia	2 (66.7)	1 (33.3)	3 (100.0)	1 (33.3)	5 (83.3%)	0	4 (80.0)	1 (20.0)
Aspartate Aminotransferase high	0	0	3 (100.0)	0	3 (50.0)	0	0	0
Glutamic‐pyruvic transaminase high	1 (33.3)	0	3 (100.0)	0	1 (16.7)	0	1 (20.0)	0
Alkaline phosphatase high	1 (33.3)	0	2 (66.7)	0	3 (50.0)	0	1 (20.0)	0
D‐dimer high	1 (33.3)	0	1 (33.3)	0	1 (16.7)	0	1 (20.0)	0
Gastrointestinal								
Nausea	0	0	1 (33.3)	0	2 (33.3)	0	0	0
Vomiting	1 (33.3)	0	1 (33.3)	0	3 (50.0)	0	1 (20.0)	0
Oral mucositis	0	0	0	0	1 (16.7)	0	0	0
Diarrhea	0	0	1 (33.3)	0	0	0	3 (60.0)	0
Abdominal pain	0	0	1 (33.3)	0	2 (33.3)	0	0	0
Constipation	0	0	0	0	3 (50.0)	0	1 (30.0)	0
Ascitic fluid		1 (33.3)						
Other adverse events								
Sinus bradycardia	0	0	0	0	4 (66.7)	0	1 (20.0)	0
Palpitation	0	0	0	0	2 (33.3)	0	0	0
Chest distress	0	0	0	0	4 (66.7)	0	0	0
Deep venous thrombosis	1 (33.3)	0	0	0	0	0	0	0
Rash	1 (33.3)	0	1 (33.3)	2 (33.3)	2 (33.3)	0	1 (20.0)	0
Interstitial lung disease	0	0	0	2 (33.3)	2 (33.3)	0	0	0
Splenic infarction	0	0	0	0	1 (16.7)	0	0	0

As depicted in Table 4, six patients (35.3%) encountered a total of 10 SAEs, with thromboembolic events noted in two patients. Notably, the C_max_ levels in these two patients were relatively lower. So these events were deemed more closely associated with gemcitabine and the advanced disease state rather than LH011. Since these SAEs occurred beyond the DLT observation period, they did not meet the DLT definition. Additionally, the number and severity of these SAEs did not demonstrate a dose‐dependent trend, likely due to the small sample size and short treatment duration.

**TABLE 4:** Serious AEs related to the study drugs.

Group	Patients with SAE	SAEs
Arm A	1	Cerebral infarction (grade 4) Upper gastrointestinal hemorrhage (grade 3)
Arm B	2	Myocardial infarction (grade 4)
		Pulmonary infection (grade 3)
Arm C	1	Sinus bradycardia (grade 1) Ventosity (grade 3)
Arm D	2	Ascitic fluid (grade 3)
		Thrombocytopenia (grade 4)
		Acute kidney injury (grade 3)
		Drug rash (grade 2)

### 3.4 Pharmacokinetics

**FIGURE 1:**
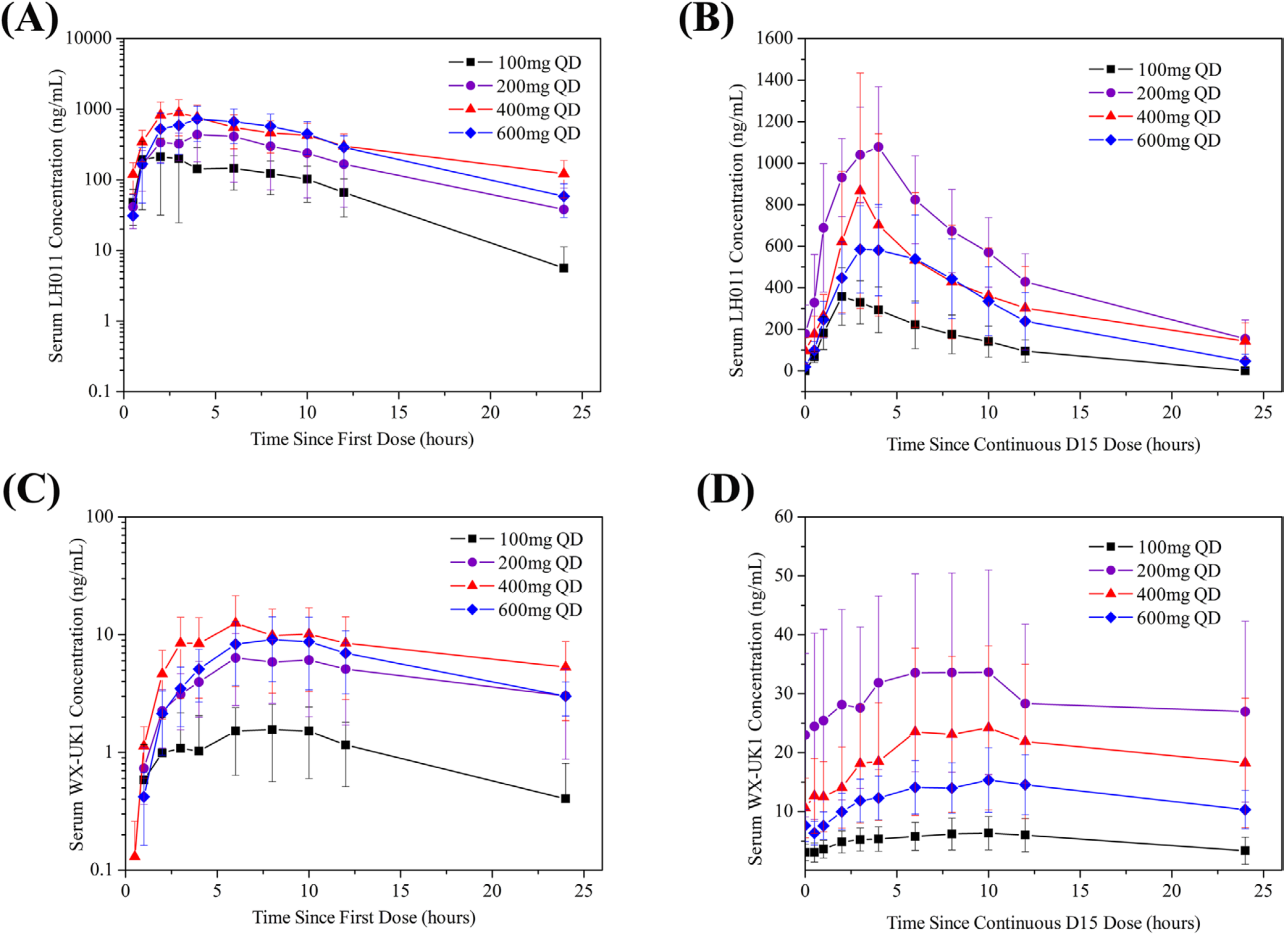
(A) Mean serum concentration profiles for LH011 after first single doses of LH011, QD, daily. (B) Mean serum concentration profiles for LH011 after continuous D15 doses of LH011, QD, daily. (C) Mean serum concentration profiles for WX‐UK1 after first single doses of LH011, QD, daily. (D) Mean serum concentration profiles for WX‐UK1 after continuous D15 doses of LH011, QD, daily.

Figure 1 illustrates the pharmacokinetic profiles of LH011 and its active metabolite WX‐UK1 after the first dose and continuous D15 dose. Corresponding pharmacokinetic parameters are summarized in Table 5. The AUC and C_max_ of LH011 and WX‐UK1, whether after the initial single dose or at steady‐state (expected to be reached by D15), demonstrated a dose‐related increase from 100 to 400 mg, with no significant change in the 600 mg group, as indicated in Table 5. Moreover, serum concentrations of LH011 and WX‐UK1 exhibited a dose‐related increase from 100 mg to 400 mg following the initial single dose. However, in the steady‐state, they displayed between‐dose variability, as depicted in Figure 1.

**TABLE 5:** Summary of serum LH011 and WX‐UK1 pharmacokinetic parameters.

Parameter		100 mg qd	200 mg qd	400 mg qd	600 mg qd
Day 1					
LH011	T_1/2, h_	5.19 ± 2.81	5.50 ± 0.67	8.50 ± 3.01	9.79 ± 9.29
	C_max, ng/ml_	279.67 ± 255.20	548.62 ± 539.85	1786.93 ± 1006.86	798.49 ± 846.53
	AUC_0–24, h*ng/ml_	1721.57 ± 1585.88	5053.37 ± 6481.69	17639.8 ± 7733.35	7210.17 ± 8247.14
WX‐UK1	T_1/2, h_	68.87 ± 81.83	11.58 ± 0^a^	18.43 ± 5.35	10.77 ± 3.84
	C_max, ng/ml_	2.47 ± 1.35	7.26 ± 7.47	25.13 ± 26.69	9.69 ± 11.53
	AUC_0–24, h*ng/ml_	33.71 ± 29.89	113.95 ± 123.98	365.4 ± 349.2	133.16 ± 152.02
Day 15					
LH011	T_1/2, h_	4.64 ± 0.77	7.48 ± 2.58	12.37 ± 4.83	4.04 ± 1.55
	C_max, ng/ml_	369.54 ± 229.98	1309.17 ± 483.64	1610.8 ± 1778.32	641.00 ± 501.84
	AUC_0–24, h*ng/ml_	2452.75 ± 1826.46	13273.94 ± 7332.88	16158.1 ± 15378.34	6580.11 ± 6783.19
WX‐UK1	T_1/2, h_	6.54 ± 0^a^	27.84 ± 13.02	46.4 ± 16.1	21.13 ± 4.65
	C_max, ng/ml_	6.63 ± 4.70	39.05 ± 32.70	45.43 ± 42.31	16.96 ± 11.44
	AUC_0–24, h*ng/ml_	120.76 ± 101.02	773.60 ± 691.11	891 ± 824.75	302.50 ± 236.35

*Note:* All data are expressed as mean ± SD.

Abbreviations: AUC_0–24_, area under the concentration‐time curve from zero to 24 h; C_max_, maximum concentration; T_1/2_, terminal half‐life.

^a^ Only one patient's data, and others were under the lower limit of quantitation(LLOQ).

LH011 was eliminated from plasma with mean terminal‐phase half‐life values (t1/2) ranging from 5.19 to 9.79 h at doses of 100–600 mg after the initial single dose. At steady‐state, t1/2 ranged from 4.64 to 12.37 h at doses of 100–400 mg, with individual differences observed at the 600 mg dose. Meanwhile, the t1/2 of WX‐UK1 exhibited between‐patient variability both after the initial single dose and at steady state.

Based on the pharmacokinetic data presented in Figure 1, both LH011 and its metabolite WX‐UK1 demonstrated a clear linear increase in the pharmacokinetic curves from 100 to 200 mg. However, from 200 to 400 mg, the curves became very similar, with some overlap. Furthermore, no DLTs were observed in the 100, 200, or 400 mg dose groups during the DLT observation period. However, SAEs occurred beyond the DLT observation window. Consequently, we decided to include three additional patients at the 400 mg dose to further validate the pharmacokinetics and its safety.

Regarding the pharmacokinetic data from the five subjects in the 600 mg dose group, neither LH011 nor its metabolite WX‐671 demonstrated a significant linear increase in the pharmacokinetic curve. In fact, the values were lower than those observed in the 400 and 200 mg dose groups, and no improvement in efficacy was noted. Based on this, the 200 and 400 mg doses may be considered the preferred dosing options. Due to these considerations, 600 mg dose level was incomplete and stopped at five instead of six patients.

### 3.5 Efficacy

**FIGURE 2:**
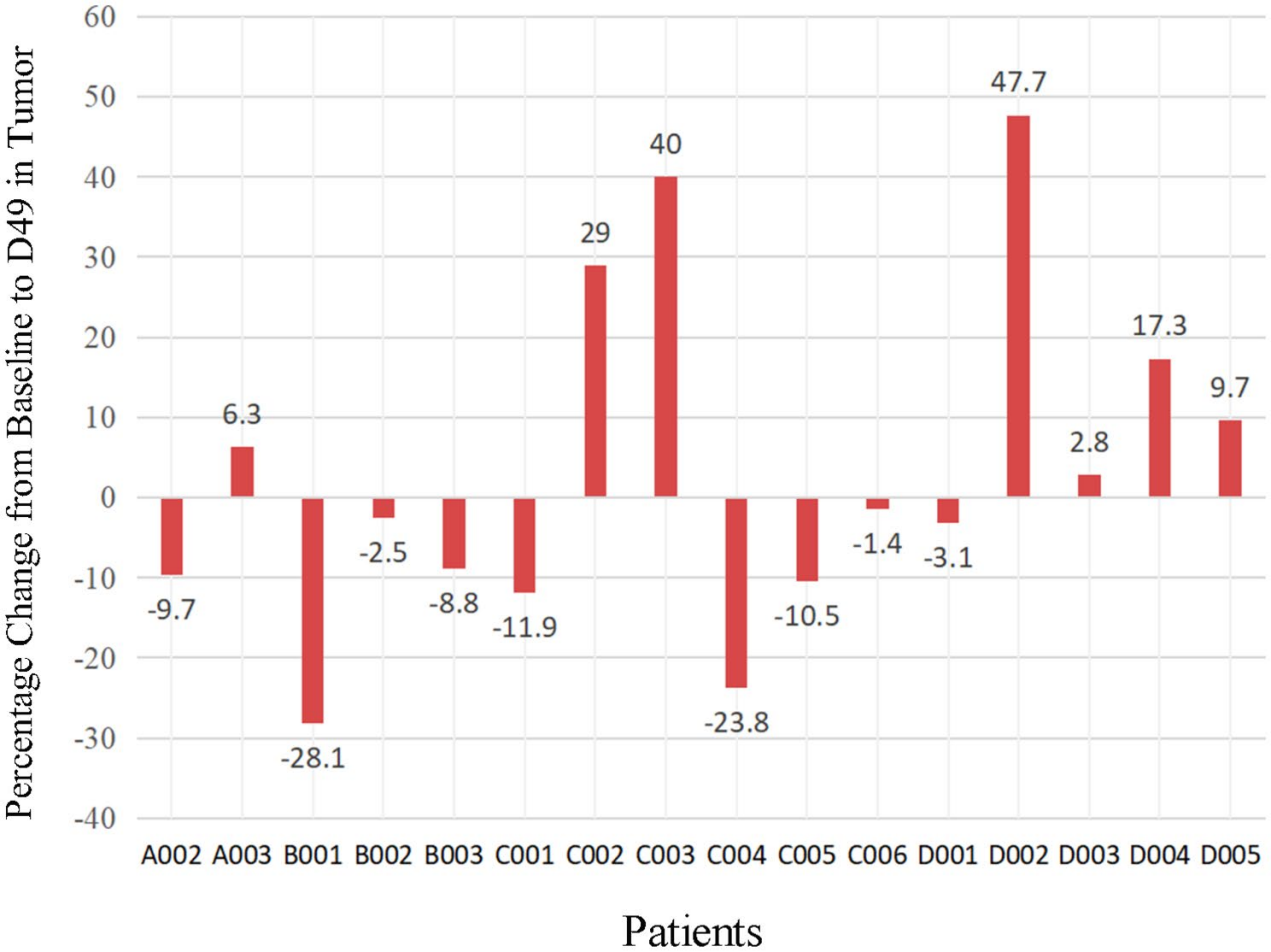
Maximum percentage change in the tumor size in patients receiving LH011 combined with 1000 mg/m^2^ of gemcitabine. A001 withdraw the study because of the SAE on D47.

**FIGURE 3:**
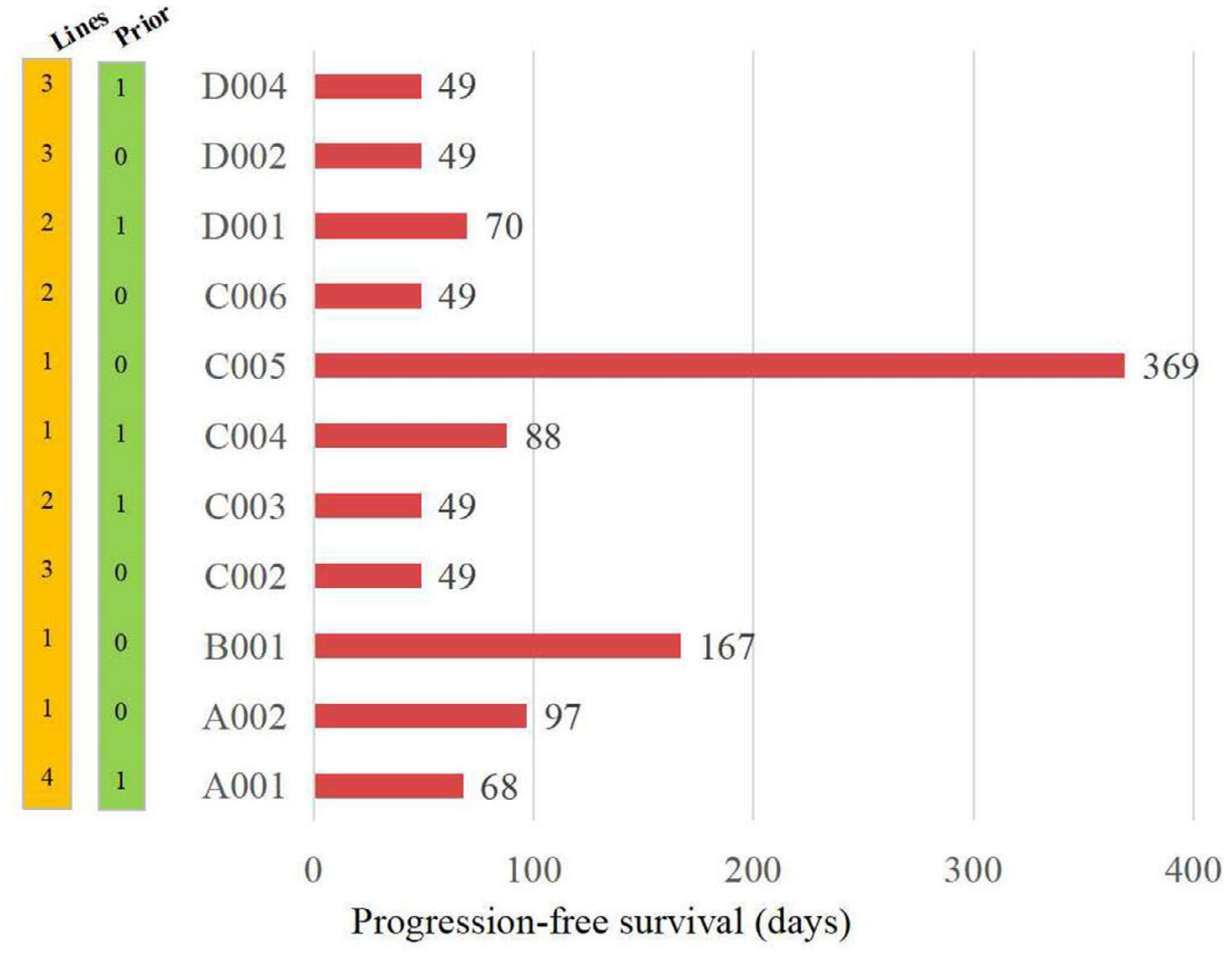
Progression‐free survival (PFS) of the patients receiving LH011 combined with 1000 mg/m2 of gemcitabine. Note for the PFS not collected: A003, D005 experienced stable disease (SD) on D49, but did not signed informed consent to continue medication. C001 withdraw the study because of the pneumonia on C4D21. B002, B003, D003 also withdraw the study because of the SAE. C005 data was collected until April 12. 2023 because the patient withdrew the study due to the recurrent sinus bradycardia.

All 17 patients had measurable disease at baseline. During the main research period (D0–D49), 12 patients (70.6%) experienced SD, four patients (23.5%) experienced PD, while one patient withdrew from the study due to a SAE on D47, resulting in no patients experiencing partial response (PR). The best percent change from baseline in target lesion size is illustrated in Figure 2, where nine patients (52.9%) experienced tumor shrinkage, including one patient with a 28.13% decrease. Treatment lines ranged from 1 to 4, with an average of the second line. Of the 17 enrolled patients, six had previously received gemcitabine. There was no significant difference in tumor efficacy observed among the four groups. Moreover, among the 12 SD patients, eight signed informed consent to continue medication. The average PFS was 100 days, with one patient achieving PR after 4 cycles and another patient reaching a PFS of 369 days (Figure 3).

**FIGURE 4:**
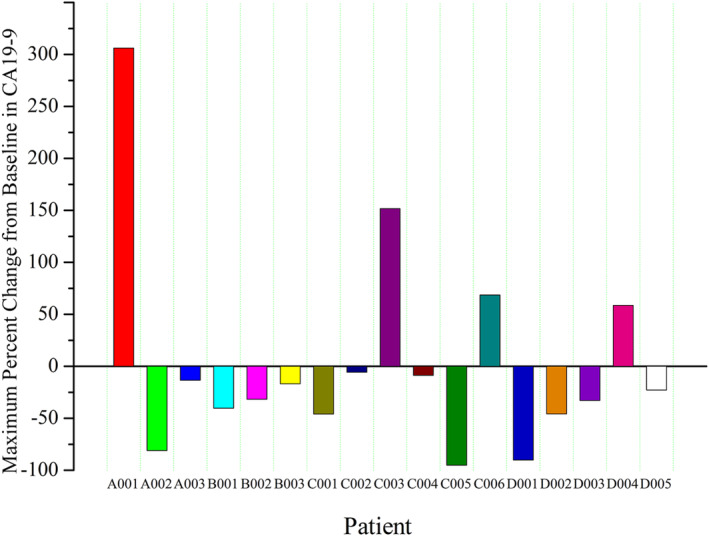
Maximum percentage change in CA19‐9 levels in patients receiving LH011 combined with 1000 mg/m^2^ of gemcitabine.

The maximum percent change in CA19‐9 level from baseline is depicted in Figure 4. Most patients experienced a decline in CA19‐9 levels, with nine patients (52.9%) experiencing ≥ 20% decline, six patients (35.3%) experiencing ≥ 40% decline, and three patients (17.6%) experiencing ≥ 80% decline. The median time to maximum decrease was on D49. Patients with significant decreases in CA19‐9 levels also showed regression in tumor diameters, with no significant difference observed among the four groups.

D‐dimer levels at baseline and D49 are summarized in Table 6. There was no significant elevation in D‐dimer levels from baseline to D49, and no significant differences were observed among the four groups.

**TABLE 6:** The D‐dimer level on baseline and the D49.

	100 mg LH011 + GEM			200 mg LH011 + GEM			400 mg LH011 + GEM						600 mg LH011 + GEM				
	A001	A002	A003	B001	B002	B003	C001	C002	C003	C004	C005	C006	D001	D002	D003	D004	D005
D0 (mg/l)	3.00	0.29	0.11	0.74	0.93	0.87	5.75	0.72	1.61	3.57	0.26	1.68	1.41	1.28	0.51	0.39	1.05
D49 (mg/l)	6.69	0.48	0.22	0.23	0.93	1.83	1.36	0.68	0.61	1.98	0.17	0.99	1.41	0.74	0.46	NA^a^	2.45
Difference	3.69	0.19	0.11	−0.51	0.00	0.96	−4.39	−0.04	−1.00	−1.59	−0.09	−0.69	0.00	−0.54	−0.05	NA	1.4
Mean	1.3			0.2			−1.3						0.2				
SD	2.0			0.7			1.6						0.83				

^a^ The data were not collected because the patient refused to return to the hospital.

## 4 Discussion

Since Billroth's seminal discovery of tumor cells in fibrinolytic thrombosis in 1878 [14], which sparked speculation about fibrin's role in tumor invasion and metastasis, numerous studies have reinforced this hypothesis. Research during the same period as Billroth's work supported the notion that fibrinogen, fibrin, and the fibrinolytic system could be implicated in tumor growth and metastasis [8]. For instance, studies like those by Kwaan and Lindholm in 2019 further highlighted this association. Additionally, investigations into the upregulated uPA‐uPAR system have revealed its correlation with tumor invasiveness and metastasis, as detailed in works by Masucci et al. in 2022 [15].

Elevated levels of uPA and uPAR have been consistently linked to increased malignant behavior and poorer patient outcomes in various cancers, such as breast cancer [16], prostate cancer [17], and others. Consequently, inhibiting the upregulated uPAR system holds promise as a therapeutic approach to mitigate the invasive and metastatic properties of various tumors. While gene therapy, drug therapy, and immunotherapy targeting the uPA‐uPAR system have been actively pursued, no drugs have yet reached the market.

Pancreatic cancer remains a challenging disease to treat, particularly in its metastatic form, underscoring the need for novel therapeutic strategies to improve patient prognosis. Urinary hormone‐type plasminogen activating factor (uPA) has emerged as a pivotal regulator of epithelial–mesenchymal transformation (EMT) and cancer metastasis, as elucidated in studies such as those by Bydoun et al. in 2018 [18] and Wu et al. in 2022 [19].

Plasminase‐induced extracellular matrix degradation and epithelial–mesenchymal cell transition, mediated by factors like matrix metalloproteinase‐14 and transforming growth factor‐β, facilitate pancreatic cancer cell invasion and metastasis [20]. High expression of urinary hormone‐type plasminogen activating factor mRNA correlates with poor overall and disease‐free survival in pancreatic cancer patients, as Professor Winter [21] described their research confirms a positive correlation between uPA serum levels and CA19‐9, as well as the negative prognostic impact of elevated uPA concentrations in pancreatic cancer patients. Preclinical studies in metastatic rat tumor models have demonstrated the efficacy of uPA inhibitors like WX‐UK1 and oral prodrug upamostat (WX‐671) in reducing tumor growth and metastasis [22, 23].

Building upon this research, our trial aims to evaluate the safety and efficacy of upamostat in combination with gemcitabine for locally advanced or metastatic pancreatic cancer. Pharmacokinetic data from the trial indicate a linear relationship between drug dosage and plasma exposure within certain dose groups. As described in previous studies by Professor Meyer [23] in the paper “The Oral Serine Protease Inhibitor WX‐671: First Experience in Patients with Advanced Head and Neck Carcinoma,” daily oral doses of 100, 200, or 400 mg of WX‐671 were administered for 15 days. Pharmacokinetic data showed that the 400 mg daily dose did not result in higher tissue concentrations of WX‐UK1 compared to the 200 mg dose. In our study, pharmacokinetic analysis similarly revealed a dose‐dependent increase in exposure to LH011 and its metabolite, WX‐UK1, from 100 to 400 mg, but not with the 600 mg dose. Furthermore, the pharmacokinetic curves for the 200 and 400 mg groups were very similar, with noticeable overlap.

Safety data reveal manageable hematological toxicities primarily attributable to gemcitabine, with sinus bradycardia possibly associated with upamostat. Notably, no dose‐limiting toxicity (DLT) events occurred across all dose groups in our study, indicating the safety and tolerability of LH011, consistent with previous findings [9, 24, 25]. Based on the combined pharmacokinetic (PK) and safety data, it suggests that either 200 mg or 400 mg may represent the optimal dose.

Gemcitabine is commonly used as a standard first‐line treatment or as a secondary option to alleviate symptoms and extend survival in patients with advanced pancreatic cancer. However, data from the ACCORD 11 trial [6] and the MPACT trial [7] show that gemcitabine monotherapy, even as a first‐line treatment, results in a progression‐free survival (PFS) of approximately 3 months and an overall survival (OS) of around 6 months. Most randomized controlled trials (RCTs) indicate that gemcitabine‐based combination therapies provide better outcomes compared to gemcitabine alone [4, 5, 6, 7], although they also carry a higher incidence of AEs.

In this study, as shown in Figure 3, only four patients received gemcitabine as first‐line therapy. Among these, the longest PFS was 1 year, the shortest was approximately 3 months, and the average PFS was around 6 months. Additionally, the CA19‐9 levels of all four patients decreased to varying degrees, suggesting that upamostat, particularly when combined with gemcitabine, demonstrates promising efficacy in stabilizing disease progression and reducing tumor burden in pancreatic cancer patients. While further research is warranted to elucidate the impact of uPA expression levels on treatment response, our study underscores the potential of targeting the uPA–uPAR system as a therapeutic strategy in pancreatic cancer management. Additionally, biomarkers like D‐dimer may serve as valuable indicators of treatment response and disease progression [26, 27, 28], warranting further investigation.

## Author Contributions

**Xiuping Lai:** data curation (equal), investigation (equal), methodology (equal), validation (equal), visualization (equal), writing – original draft (equal). **Di Cheng:** conceptualization (equal), data curation (equal), formal analysis (equal), visualization (equal), writing – review and editing (equal). **Huixin Xu:** conceptualization (equal), formal analysis (equal), investigation (equal), writing – review and editing (equal). **Jingshu Wang:** formal analysis (equal), investigation (equal), methodology (equal), writing – review and editing (equal). **Xiaozhi Lv:** formal analysis (equal), investigation (equal), methodology (equal), writing – review and editing (equal). **Herui Yao:** funding acquisition (equal), project administration (equal), resources (equal), supervision (equal), validation (equal). **Liuning Li:** investigation (equal), methodology (equal), resources (equal), visualization (equal), writing – review and editing (equal). **Junyan Wu:** conceptualization (equal), funding acquisition (equal), project administration (equal), resources (equal), supervision (equal). **Suiwen Ye:** project administration (equal), resources (equal), supervision (equal), validation (equal). **Zhihua Li:** conceptualization (equal), data curation (equal), formal analysis (equal), investigation (equal), methodology (equal).

## Ethics Statement

The studies involving human participants were reviewed and approved by the Ethical Committee of Sun Yat‐sen University Memorial Hospital.

## Consent

All patients/participants provided their written informed consent to participate in this study.

## Conflicts of Interest

The authors declare no conflicts of interest.

## Data Availability

The data underlying this article cannot be shared publicly for the privacy of individuals who participated in the study. The data will be shared on reasonable request to the corresponding author.
